# miR-424 Promotes Bovine Adipogenesis Through an Unconventional Post-Transcriptional Regulation of *STK11*

**DOI:** 10.3389/fgene.2020.00145

**Published:** 2020-03-04

**Authors:** Li Wang, Song Zhang, Wenzhen Zhang, Gong Cheng, Rajwali Khan, Zainaguli Junjvlieke, Shijun Li, Linsen Zan

**Affiliations:** ^1^ College of Animal Science and Technology, Northwest A&F University, Yangling, China; ^2^ National Beef Cattle Improvement Center, Northwest A&F University, Yangling, China

**Keywords:** bovine, preadipocytes, miR-424, *STK11*, adipogenesis, post-transcriptional regulation

## Abstract

Adipose tissue is the largest energy reservoir and secretory organ in the animal body, and is essential for maintaining normal physiological functions and metabolic balance. MicroRNAs regulate the process of adipogenic differentiation through post-transcriptional regulatory mechanisms. In the present study, miR-424 was upregulated during bovine adipocyte differentiation both *in vivo* and *in vitro*. The overexpression and interference of miR-424 exhibited the positive regulatory role in the differentiation of bovine adipocytes. Furthermore, miR-424 directly binds to the three prime untranslated region (3' UTR) of serine/threonine kinase 11 (*STK11*, also called *LKB1*), a master upstream gene in the AMP-activated protein kinase (AMPK) cascade, and up-regulates its expression. Functional studies showed that the knockdown of *STK11* attenuated the pro-adipogenic effect of miR-424. Post-transcriptional regulation of *STK11* by miR-424 was mediated potentially in an RNA binding protein (RBP) binding site-dependent manner. In conclusion, our study shows that miR-424 promotes bovine adipogenesis through an unconventional post-transcriptional regulation of *STK11*, which may serve as a potential target for the regulation of bovine adipogenesis and the improvement of livestock breeding efficiency.

## Introduction

Adipose tissue is the main storage site for surplus energy, which is stored in the form of neutral triglycerides, and viewed as a biofuel reservoir that plays a crucial role in maintaining energy homeostasis (Sethi and Vidal-Puig, 2007). Adipocytes are the primary cell type in adipose tissue that deposit lipid droplets in response to overnutrition through the lipogenic pathway and release lipids during energy deficiency through the lipolytic pathway (Rutkowski et al., 2015). Lipid accumulation is not only highly correlated with metabolic disorders such as insulin resistance and type 2 diabetes mellitus (T2DM) (Greenberg et al., 2011), but also affects livestock production (Wood et al., 2008; Hausman et al., 2009). The intramuscular fat content in beef positively influences sensory quality traits and directly determines the meat grade (Hocquette et al., 2010; Park et al., 2018). Therefore, improving the fat content in beef is an essential goal of modern beef cattle breeding programs. Fat deposition and energy metabolism are regulated by multiple factors in the adipose tissue (Luo and Liu, 2016).

MicroRNAs (miRNAs), a class of small non-coding RNAs, regulate many physiological processes, including fat differentiation and development (Engin, 2017). High-throughput expression data from multiple studies have shown that miR-424 is differentially expressed during adipogenic differentiation (Ortega et al., 2010; Shi et al., 2016). The analysis of microarray data enlisting miRNA expression showed that miR-424 is strongly downregulated during the differentiation of human subcutaneous adipocytes (Ortega et al., 2010), whereas it is markedly upregulated during the differentiation of human adipose derived mesenchymal stem cells (hMSC-Ad) and human stromal vascular cells (SVCs) into mature adipocytes (Shi et al., 2016). Furthermore, we re-analyzed previously published dataset GSE48569 (Sun et al., 2014) deposited in the Gene Expression Omnibus (GEO) database, and found that the expression of miR-424 was significantly higher in the backfat tissues of adult cattle than that of fetal cattle. Although the altered expression of miR-424 during adipogenesis can be profound, the function of miR-424 in adipogenesis has not yet been determined.

The classical view is that miRNAs inhibit the expression of protein-coding genes through post-transcriptional regulation. This negative regulatory mechanism relies on the binding of miRNAs to the three prime untranslated region (3' UTR) of the target messenger RNAs (mRNAs) (Dykxhoorn et al., 2003). Several studies have revealed that miRNAs also have the capability of activating gene expression, directly or indirectly, in the presence of co-factors in various cell types (Vasudevan, 2012; Dragomir et al., 2018), and in response to different conditions (Bhattacharyya et al., 2006; Truesdell et al., 2012; Valinezhad Orang et al., 2014). RNA binding proteins (RBPs) are one of the factors involved in miRNA mediated regulation of gene expression (Gardiner et al., 2015; Connerty et al., 2016). AU-rich elements (AREs) and GU-rich elements (GREs) are sequence motifs found in the 3' UTR of many mRNAs, that upon interaction with RBPs induce the stabilization or destabilization of mRNA (Vlasova-St. Louis and Bohjanen, 2014; Fallmann et al., 2016). To date, mostly the protein-coding genes are known to be regulated by these mechanisms and the 3' UTR binding has been shown to regulate the half-life of mRNA (Vlasova et al., 2008; Halees et al., 2011; Fallmann et al., 2016). miRNAs and RBPs are considered as the two great classes of post-transcriptional regulatory molecules that alter the expression of target genes (Ciafre and Galardi, 2013). They may modulate the abundance of the target transcripts independently or coordinately through complex regulatory networks (Jiang and Coller, 2012).

In the current study, we evaluated miR-424 as a potential candidate for regulating bovine adipose differentiation, and identified serine/threonine kinase 11 (*STK11*, also called *LKB1*) as its atypical target. Furthermore, *in vitro* experiments were performed to investigate the regulation of the miR-424/*STK11* axis during bovine preadipocytes differentiation. We further explored the unconventional mechanism of post-transcriptional up-regulation of the *STK11* gene by miR-424.

## Materials and Methods

### Ethical Statement

Animal samples, used in this study, were collected using a protocol approved by the “Animal Care and Use Committee” of the College of Animal Science and Technology, Northwest A&F University (No. NWAFAC1117).

### Sample Collection

All the Qinchuan cattle used in this study had similar genetic background, and animals were housed under similar management, nutritional, and environmental conditions at the experiment farm of the National Beef Cattle Improvement Center (NBCIC, Yangling, China). At each developmental stage (0, 6, 12, 18, 24, and 60 months of age), three cattle were randomly selected to collect the dorsal subcutaneous adipose tissue (backfat), which is deposited between the 12th and 13th rib. The adipose tissue was collected after animals were slaughtered from the mechanized line at the Shaanxi Qinbao Animal Husbandry Development Co. Ltd. In addition, perirenal and groin fat tissue samples were collected from the newborn calves. All samples were immediately stocked in liquid nitrogen and subsequently stored at −80°C until RNA isolation.

### Cell Culture

HEK293A cell line was purchased from the American Type Culture Collection (ATCC, Manassas, VA, USA). The bovine primary preadipocytes were isolated from the Qinchuan cattle and maintained at NBCIC (Wang Y. et al., 2018). HEK293A cells and bovine preadipocytes were cultured in DMEM-high glucose and DMEM-F12 (HyClone, Logan, UT, USA) medium, respectively, supplemented with 10% fetal bovine serum (FBS, Gibico, Carlsbad, CA, USA) and 1% penicillin-streptomycin (HyClone). The cells were incubated at 37°C under saturated humidity and 5% CO_2_.

### Transfection

The bovine preadipocytes were transfected at 70–80% confluence with miR-424 mimics (50 nM) or miR-424 inhibitor (100 nM) or their respective negative control (NC) (RiboBio, Guangzhou, China). Co-transfection was performed with *STK11* 3' UTR related plasmid (100 ng/μl) using Lipofectamine 3000 (Invitrogen, Carlsbad, CA, USA) reagent according to the manufacturer’s instructions.

### Recombinant Adenovirus Production and Infection

The short hairpin RNA (shRNA) targeting *STK11* was designed and synthesized as follows: sense, 5'-GACGACGAGCTCTTCGACATTTCAAGAGAATGTCGAAGAGCTCGTCGTCTTTTTTAGATCTG-3' and antisense, 5'-GATCCAGATCTAAAAAAGACGACGAGCTCTTCGACATTCTCTTGAAATGTCGAAGAGCTCGTCGTC-3'. The recombinant adenovirus pAd-sh-*STK11* was constructed using AdMax^TM^ recombinant adenovirus packaging system, as previously described (Zhang et al., 2019). The virus titer was detected by enhanced green fluorescent protein (EGFP) labeled method (Le et al., 2004). An empty adenovirus vector (pAd-EGFP) was used as the control. The recombinant adenoviruses were infected at optimal multiplicity of infection (MOI) value into bovine preadipocytes, and all samples were collected 48 h post-infection for quantitative RT-PCR (qRT-PCR) or western blot assay.

### Adipogenic Differentiation and Lipid Droplet Staining

Bovine preadipocytes were differentiated as described previously (Wang et al., 2018). Following differentiation for 4 or 8 days, the cells were visually analyzed using BODIPY or Oil red O (ORO) staining of the lipid droplets (LDs), respectively. For the ORO staining, the cells were washed 3 times with PBS and fixed with 4% paraformaldehyde for 30 min. They were stained with ORO solution (0.3% ORO, 60% isopropanol, and 40% PBS) for 30 min in the dark. For the BODIPY staining, the cells were incubated for 30 min after fixation, in a 1:1,000 dilution of 1 mg/ml BODIPY 493/503 (Invitrogen) in PBS at room temperature. We used 4,6-diamidino-2-phenylindole (DAPI) to identify the nuclei. Images were visualized with a Live Cell Imaging System (Nikon Instruments, Europe BV, Kingston, Surrey, England). Lipid accumulation was quantified from the fluorescence intensity parameter of the BODIPY dye (green fluorescence) using ImageJ software.

### RNA Isolation and Quantitative Real-Time PCR Assay

Total RNA was isolated from tissues and cells using RNAiso regent (Takara, Dalian, China). The qRT-PCR experiments were performed with the ABI7500 Fast Real-Time PCR System (Applied Biosystems, Foster City, CA, USA). Expression of miR-424 was quantified using the miRcute Plus miRNA First-Strand cDNA Kit and miRcute Plus miRNA qPCR Kit (SYBR Green) (Tiangen, Beijing, China). The mRNAs were quantified using PrimeScript^TM^ RT Reagent Kit with gDNA Eraser and SYBR Premix Ex Taq^TM^ II Kit (Takara); 2^−∆∆Ct^ method was used to determine the relative mRNA and miRNA expression levels as described earlier (Livak and Schmittgen, 2001). The genes GAPDH and ACTB (for mRNA) or RPS18 and U6 (for miRNA) were used as internal controls for qRT-PCR. The experiments were performed in three biological and technical replicates for each assay. The specific primer sets used in the qRT-PCR are listed in **Table S1**.

### Protein Extraction and Western Blot Analysis

Cells were lysed in radioimmunoprecipitation assay (RIPA) lysis buffer supplemented with proteinase inhibitors. An equal amount of total protein for each sample was separated on a 12% sodium dodecyl sulfate-polyacrylamide gel electrophoresis (SDS-PAGE) following conventional western blotting procedures as previously described (Bass et al., 2017). Primary antibodies against STK11 (1:1,000 (LS−C388827, Lifespan Biosciences, Seattle, USA)), β-actin (1:5,000) (NB100-56874, Novus, Littleton, USA), and horseradish peroxidase (HRP)-conjugated secondary antibody (1:5,000) (D110056, Sangon, Shanghai, China) were used. The protein bands were visualized using Gel DocTM XR+ Gel Documentation System (Bio-Rad, Hercules, CA). Band intensity was quantified using the ImageJ software.

### 3′ Rapid Amplification of Complementary DNA Ends

Total RNA was isolated from the bovine perirenal and groin fat tissues and then reverse transcribed using the "hybrid" primer (QT). The first round of amplification was performed using *STK11* gene-specific outer primer (GSP1) and a unique oligonucleotide sequence (Qo), and the second round of amplification cycle was carried out using a gene-specific inner primer (GSP2) and "nested" primers (QI) to quench the amplification of nonspecific products. Finally, all the PCR products were cloned into a pMD19-T vector (Takara) and sequenced. The 3' UTR sequences are listed in **Figure S1**. All the primers used in this method are listed in **Table S1**.

### Reporter Plasmid Construction and Luciferase Assay

A fragment of 3' UTR from cattle *STK11* transcript, containing the predicted miR-424 recognition element (MRE) or the potential RBP binding sites (AREs and GREs), were cloned into a psi-CHECK2 vector. Further, the miR-424 predicted target site (UGCUGCU) was mutated to CAUCAUG. All plasmids were verified by sequencing. Sequence information for the primers is listed in **Table S1**.

For the luciferase reporter assay, cells at 60% confluence in 24-well plates were transfected using Lipofectamine 3000 reagent. Co-transfections were performed with 100 ng of empty, or wild type or mutant *STK11* 3' UTR luciferase plasmids, and 50 nM miR-424 mimics or NC mimics per well (in triplicates). Empty vectors were used as negative controls. After 36 h, the cells were collected, lysed, and assayed for luciferase activity using the Dual-Luciferase Reporter Assay System (Promega, Madison, WI, USA) according to the manufacturer’s instructions. Firefly luciferase activity was used as an internal reference standard.

### Statistical Analyses

All data are presented as the mean ± SEM and analyzed with GraphPad Prism 7 software. Two groups were compared using the unpaired two-tailed Student’s t-test. A value of *p* < 0.05 was considered to be statistically significant.

## Results

### miR-424 Is Upregulated During Bovine Adipogenesis

**Figure 1:**
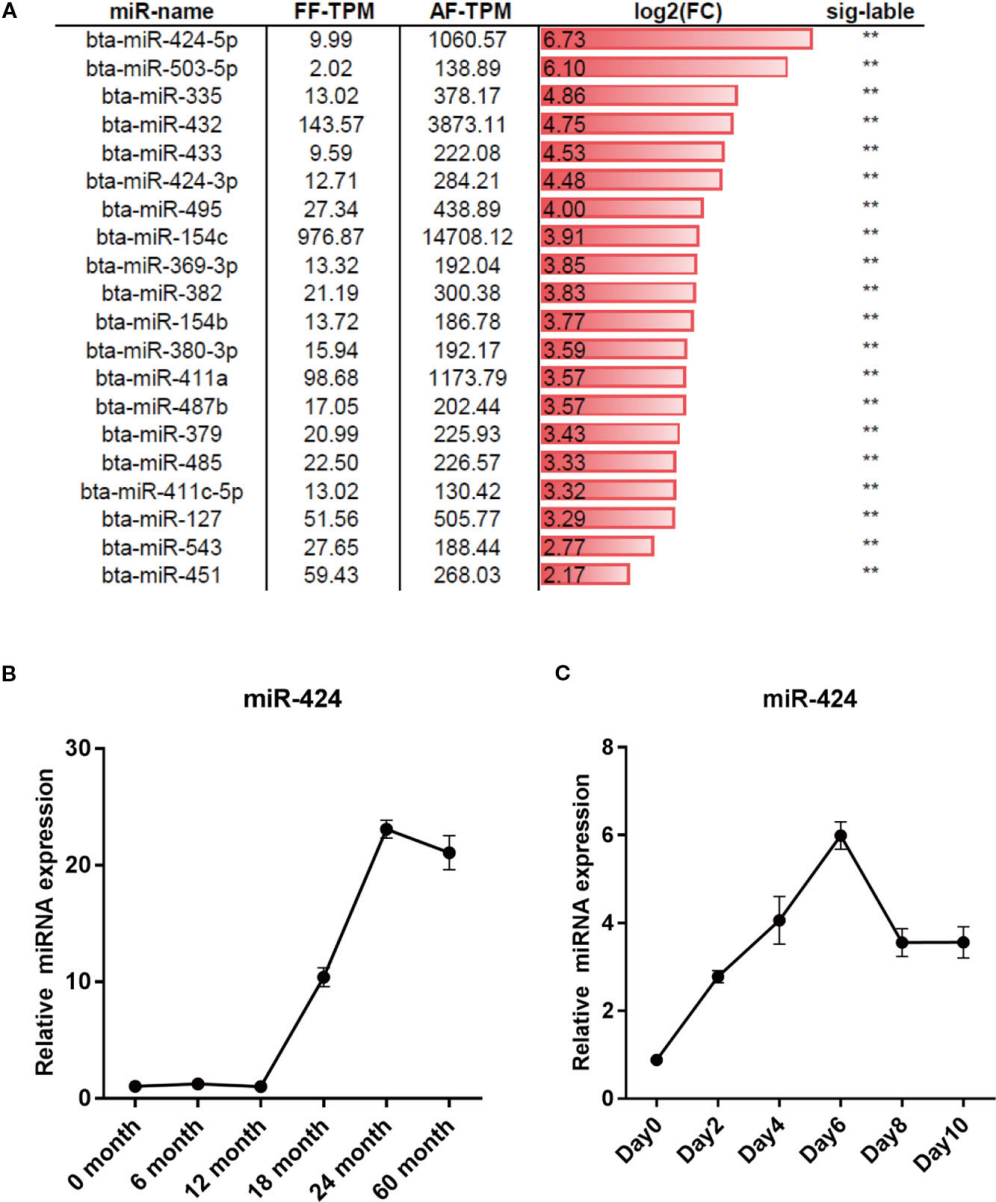
miR-424 is up-regulated during bovine adipogenesis. **(A)** Differentially expressed mircroRNAs (miRNAs) (DEMs) between the fetal and adult stages of backfat tissues in Qinchuan cattle were obtained in dataset GSE48569 based on the value of transcripts per million (TPM). The top 20 significantly upregulated miRNAs in the backfat of adult Qinchuan cattle (AF-TPM) compared to that of fetal Qinchuan cattle (FF-TPM) are listed and sorted by log_2_ based fold-change (log2FC). Sig-label shows significance. **(B, C)** qRT-PCR assays of miR-424 expression during the process of growth (0–60 months old) of Qinchuan cattle backfat and the process of differentiation in Qinchuan cattle preadipocytes. Results are shown as the mean ± SEM and the data are representative of three biological and technical replicates. **p <  0.01.

To screen for novel miRNAs regulating bovine adipogenesis, we re-analyzed the dataset GSE48569 from the GEO database (Sun et al., 2014). The differentially expressed miRNAs (DEMs) in backfat tissues between fetal (GSM1181371) and adult (GSM1181372) stages of Qinchuan cattle were obtained based on the value of transcripts per million (TPM). Compared to the fetal, the adult had 27 upregulated miRNAs and 28 downregulated miRNAs. We focused on miR-424 (unless otherwise stated, miR-424 stands for bta-miR-424-5p in this manuscript) because of its higher differential expression (**Figure 1A**). The results of qRT-PCR assay confirmed that the expression of miR-424 was noticeably increased between 12 and 60 months of age in the backfat tissues of Qinchuan cattle (**Figure 1B**). In addition, the bovine preadipocytes were differentiated into lipid-filled adipocytes in vitro. The qRT-PCR result showed that the expression of miR-424 increased gradually during the differentiation process (**Figure 1C**). Collectively, these results indicate that miR-424 is up-regulated during bovine adipogenesis *in vivo* and *in vitro*. Thus, miR-424 appears to be involved in the regulation of bovine adipogenesis and was selected as the candidate miRNA for subsequent experiments.

### miR-424 Facilitates the Differentiation of Bovine Preadipocytes

**Figure 2:**
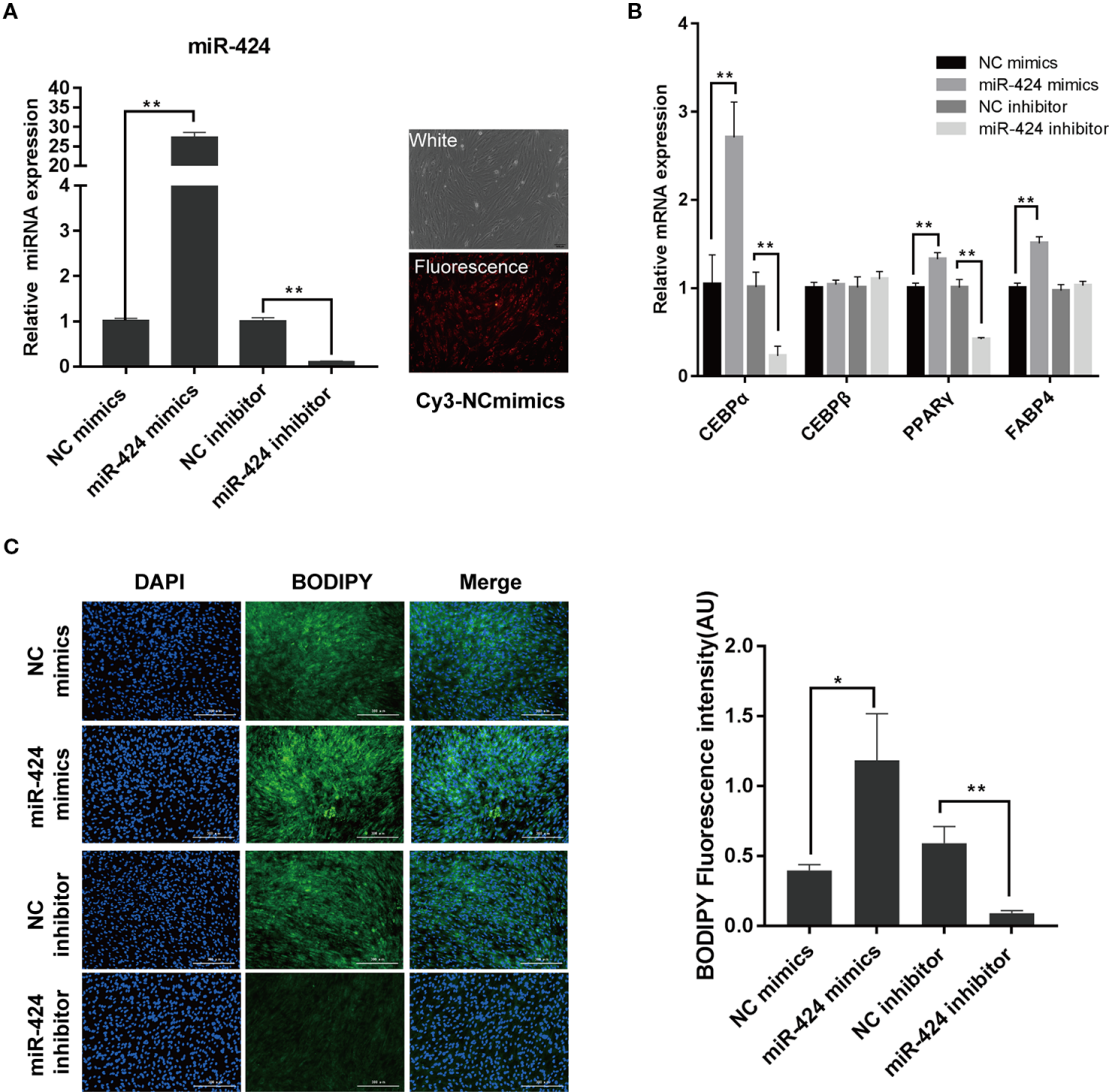
miR-424 promotes the differentiation of bovine preadipocytes. Bovine preadipocytes were transfected with miR-424 mimics, inhibitor or their respective negative control (NC) and induced differentiated into mature adipocytes 60 h post-transfection. **(A)** The transfection efficiency was determined 48 h after transfection using quantitative real-time (qRT)-PCR (left), or Cy3-labeled NC mimics (right), bar, 100 µm. **(B)** The messenger RNA (mRNA) levels of the adipogenic markers were measured by qRT-PCR on day 3 of differentiation. **(C)** Representative images of BODIPY 493/503 staining for lipids (green) in bovine preadipocytes on day 4 of differentiation. Nuclei were stained with 4,6-diamidino-2-phenylindole (DAPI) (blue). Bar, 300 μm. Fluorescence intensity of the BODIPY signal (arbitrary units, in %) was analyzed by the ImageJ tool. Results are shown as the mean ± SEM and the data are representative of three biological and technical replicates. *p <  0.05, **p <  0.01.

To understand the regulation and functional activity of miR-424 during adipocyte differentiation, bovine preadipocytes were transfected with either miR-424 mimics, inhibitor, or their respective negative controls. The transfection efficiency was monitored using Cy3-labeled NC mimics and further confirmed by qRT-PCR assay (**Figure 2A**). Next, the cells were induced to differentiate *in vitro*. On day 3, the mRNA expression of known early markers of differentiation such as *CEBP/α, CEBP/β, PPARγ,* and *FABP4*, were analyzed (**Figure 2B**). We found that overexpression of miR-424 markedly increased the expression of *CEBP/α, PPARγ,* and *FABP4*, whereas miR-424 inhibitor significantly reduced the expression of *CEBP/α* and *PPARγ*. Interestingly, miR-424 failed to influence the expression of *CEBP/β*. Furthermore, we assessed the cellular accumulation of LDs on day 4 of differentiation by staining with BODIPY 493/503 and found that the fluorescence signal of BODIPY was notably increased in cells treated with miR-424 mimics when compared with NC mimics. Conversely, the accumulation of LDs in cells transfected with miR-424 inhibitor showed the opposite trend (**Figure 2C**). These results demonstrate that miR-424 promotes the differentiation of bovine preadipocytes.

### *STK11* Is a Direct Target of miR-424

**Figure 3:**
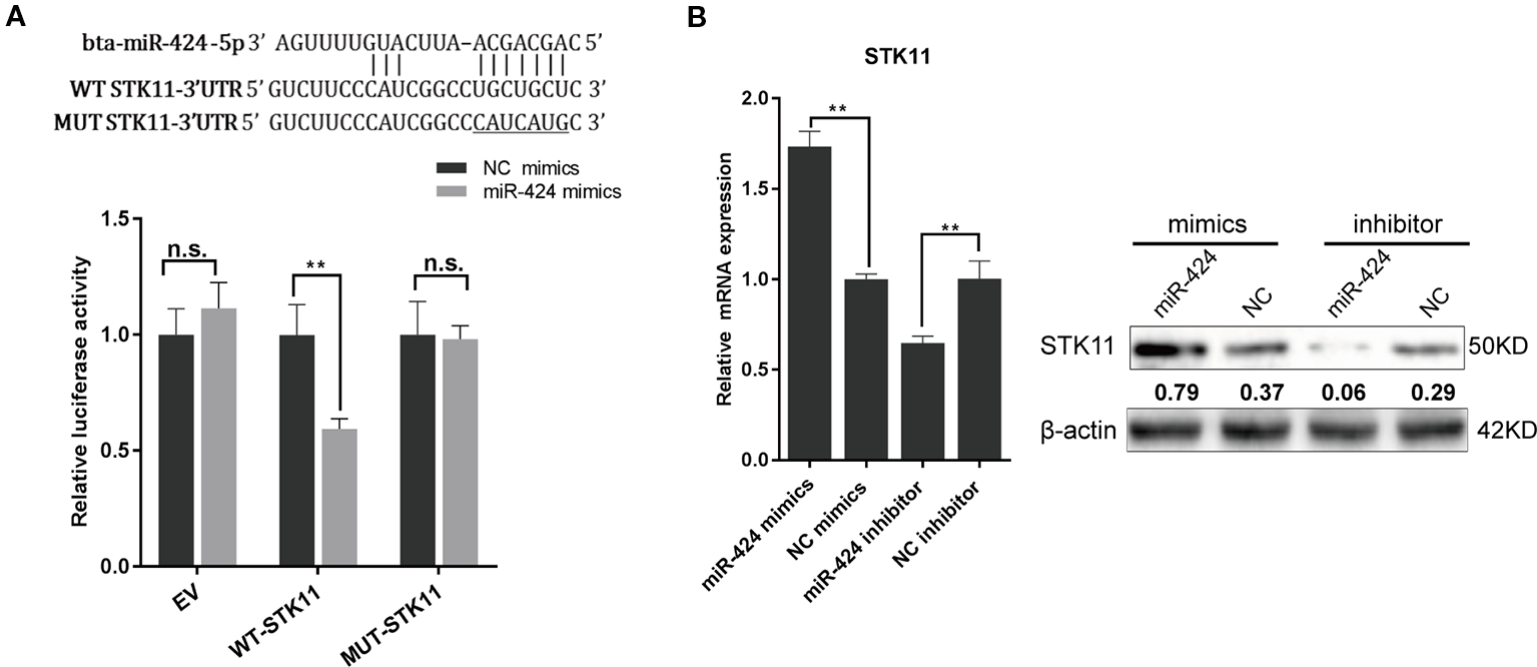
*STK11* is a direct target of miR-424 and is positively regulated by miR-424 in bovine preadipocytes. **(A)** (Top) Schematic representation of the sequence of mature miR-424. The putative binding sites were located in three prime untranslated region (3' UTR) of the bovine *STK11* gene, and the point mutation generated is underlined. (Bottom) Relative luciferase activity was measured in HEK293A cells co-transfected with the psi-Check2 empty vector (EV), *STK11* 3' UTR-WT, or *STK11* 3' UTR-MUT reporter plasmids along with NC mimics or miR-424 mimics. Renilla luciferase activity was normalized to firefly luciferase. **(B)** The messenger RNA (mRNA) and protein expression of *STK11* in bovine preadipocytes were examined 48 h post-transfection with miR-424 mimics and inhibitor. The quantitative representation of the gray ratio of STK11/β-actin protein is displayed numerically under the strip. Results are shown as the mean ± SEM and the data are representative of three biological and technical replicates. *p <  0.05, **p <  0.01, n.s., not significant.

To understand the molecular mechanism through which miR-424 promotes bovine adipogenesis, we used three softwares (TargetScan/Miranda/Mireap) to identify its putative targets that are involved in the regulation of adipocytes differentiation. *STK11*, a master upstream kinase in the AMPK cascade, was consistently screened out as a potential target of miR-424. Next, we constructed the wild-type and mutant luciferase reporter vectors for *STK11*-miR-424-MRE and performed dual-luciferase reporter assay. The results showed that miR-424 significantly suppressed the activity of wild-type reporter, while it did not affect that of the mutant reporter (**Figure 3A**). This assay confirmed that miR-424 binds to the 3' UTR of bovine *STK11*.

To gain insights into the direct effect of miR-424 on *STK11* gene expression, we examined the levels of *STK11* mRNA and endogenous protein levels 48 h post-transfection. Unexpectedly, as shown in **Figure 3B**, the *STK11* expression level increased following the treatment with miR-424 mimics, while it decreased following the treatment with miR-424 inhibitor. These results suggest that the positive regulatory effect of miR-424 on *STK11* is not through the conventional process of promoting mRNA degradation or blocking mRNA translation.

### Knockdown of *STK11* Attenuated the Bovine Preadipocytes Differentiation Induced by miR-424

**Figure 4:**
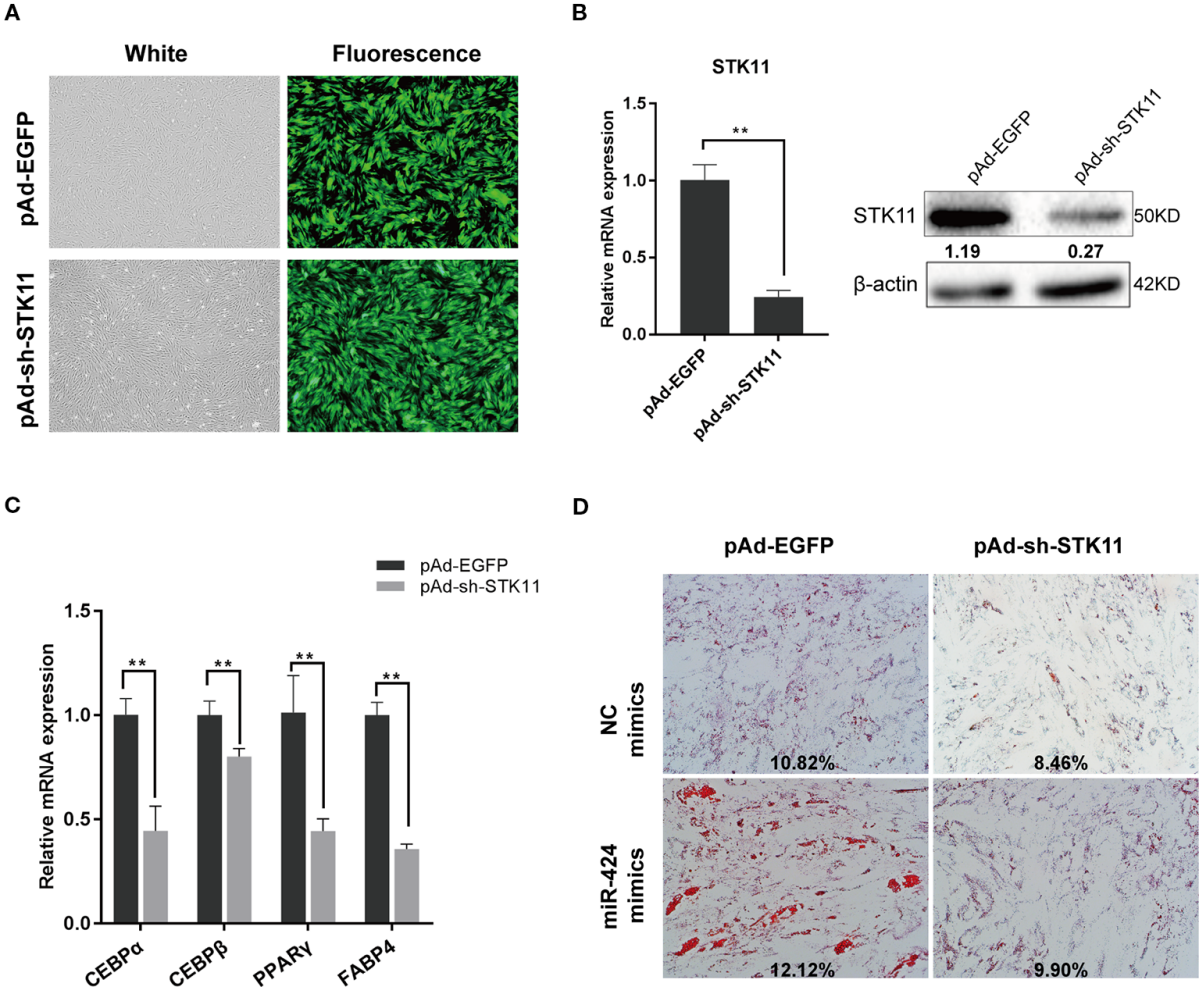
Knockdown of *STK11* attenuates the ability of bovine preadipocytes differentiation induced by miR-424. Bovine preadipocytes were infected with pAd-sh-*STK11* or pAd-EGFP recombinant adenovirus at an MOI of 50 or 60 and then differentiated into mature adipocytes. **(A)** The enhanced green fluorescent protein (EGFP) expression, considered as a qualitative indicator, was analyzed 48 h after infection to assess the infection efficiency. Bar, 200 µm. **(B)** Total mRNA and protein were extracted 48 h later, and the level of *STK11* expression was assessed by quantitative real-time (qRT)-PCR and western blotting. Relative band quantification of the gray ratio of STK11/β-actin protein was determined by the ImageJ tool (shown below the strip). **(C)** The mRNA levels of adipogenic markers were measured by qRT-PCR on day 3 of differentiation. **(D)** NC mimics or miR-424 mimics were transfected into bovine preadipocytes, and after 48 h, the cells were infected with indicated adenovirus and adipogenic differentiation was induced. The LDs content was measured on day 8 by ORO staining. Bar, 50 µm. The percentage of ORO stained area in each microscopic field was quantified using the ImageJ software. Results are shown as the mean ± SEM and the data are representative of three biological and technical replicates. **p <  0.01.

To directly validate the role of *STK11* in the pro-adipogenic effect of miR-424 in bovine preadipocytes differentiation, the cells were infected with adenovirus pAd-sh-*STK11* and pAd-EGFP, and the green fluorescent protein was analyzed 48 h post-infection to assess the infection efficiency (**Figure 4A**). As expected, specifically knockdown endogenous *STK11,* using pAd-sh-*STK11,* significantly decreased the levels of *STK11* mRNA and protein expression as compared to the pAd-EGFP (**Figure 4B**). The mRNA levels of the adipogenic markers were measured by qRT-PCR on day 3 of differentiation. We found that these genes (*CEBP/α, PPARγ,* and *FABP4*) were downregulated in response to miR-424 overexpression (**Figure 4C**), suggesting that the knockdown of *STK11* blocked bovine preadipocytes differentiation. This argument was further supported by the fact that knockdown of *STK11* expression led to an impaired accumulation of LDs in the mature adipocytes (**Figure 4D**).

Next, we investigated whether suppressing the expression of *STK11* could weaken the effects of miR-424 in preadipocytes differentiation. To address this, we performed a “rescue” assay. As illustrated by the ORO staining results (**Figure 4D**), there was a discernible difference in the accumulation of LDs in the mature adipocytes following the enforced expression of miR-424, while miR-424 could not stimulate adipogenesis in the *STK11* knockdown cells. Therefore, we concluded that miR-424 specifically promotes *STK11* expression, which in turn contributes (at least in part) to the regulatory role of miR-424 in adipocyte differentiation.

### Upregulation of *STK11* by miR-424 Occurs in a Potential RBP Binding Site-Dependent Manner

Given the positive effects of miR-424 on *STK11* mRNA and protein expression as described above, we reasoned that miR-424 might unconventionally regulate the expression of *STK11*. Several studies have revealed that the post-transcriptional upregulation by miRNAs is selective, based on the target mRNA specific sequences, and associated with RBPs (Vasudevan et al., 2007). It is not clear whether the observed role of miR-424 in positively mediating *STK11* expression is due to direct interaction with specific *STK11* mRNA sequence, or indirect interaction through unknown RBPs, or both.

**Figure 5:**
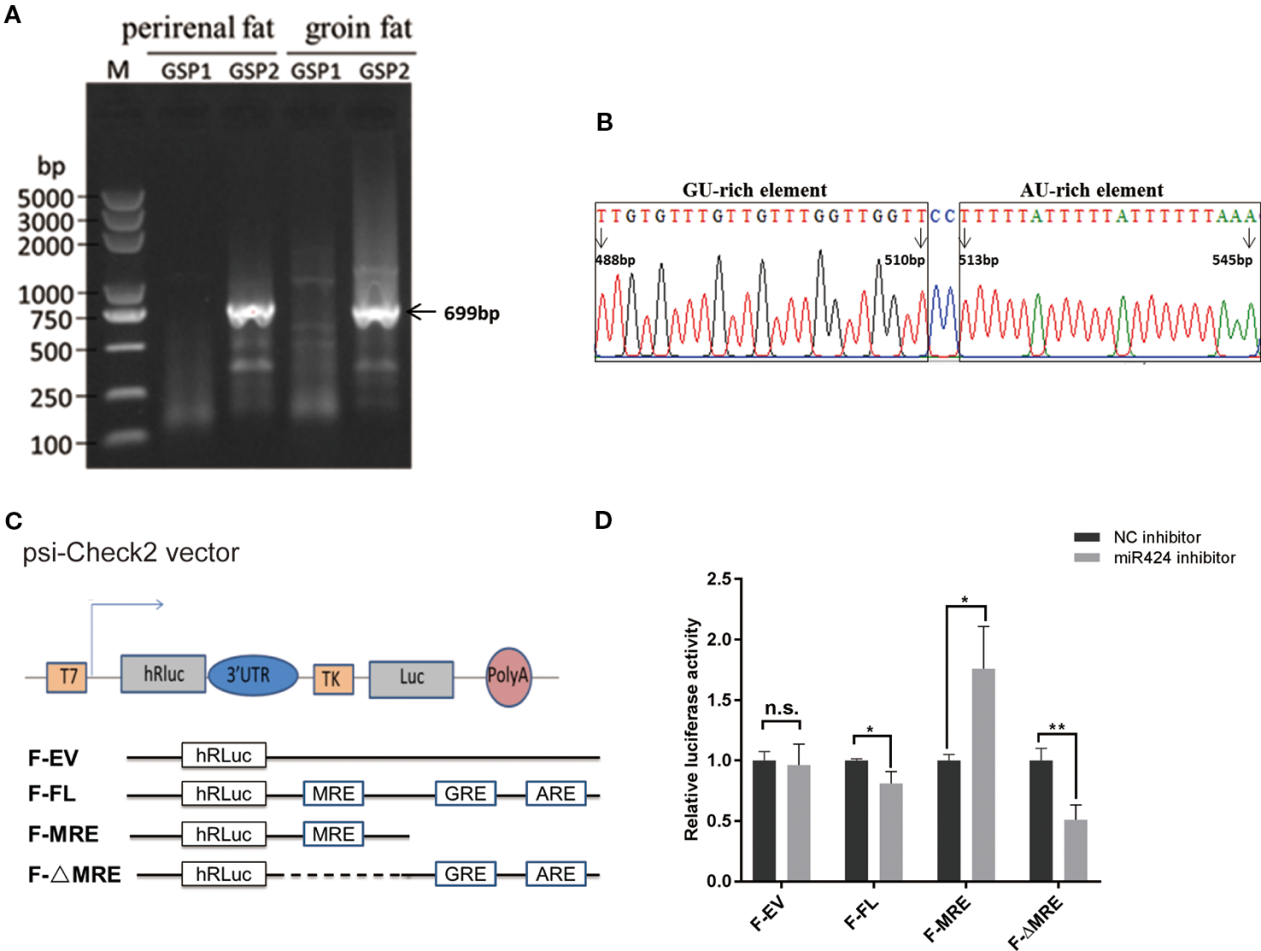
Positive regulation of *STK11* by miR-424 may be mediated by potential RBP binding sites in the three prime untranslated region (3' UTR) of *STK11*. **(A)** 3′ Rapid amplification of complementary DNA (cDNA) ends (3' RACE) derived 3' ends of the 699 bp *STK11* transcript. The complementary DNA (cDNA) were synthesized from bovine perirenal and groin fat tissues. M: DL5000 marker, the gene-specific outer primer (GSP1) lane shows the products of the first-round of PCR, and the gene-specific inner primer (GSP2) lane shows the products of the second-round of PCR. **(B)** Diagram for the distribution of potential GU-rich elements (GREs) and AU-rich elements (AREs) in 3' UTR of bovine *STK11*transcript. The first base after the termination codon (UGA) is defined as “1 bp.” **(C)** Structures of the reporter constructs used. The luciferase reporter constructs fused to the full-length *STK11* 3' UTR (F-FL), or containing the miR-424 MRE only (F-MRE), or the miR-424 MRE deleted from the full-length 3' UTR (F-ΔMRE). Potential GREs and AREs are indicated. **(D)** Bovine preadipocytes were co-transfected with 3' UTR reporter constructs and miR-424 inhibitor or NC inhibitor for 48 h. Luciferase activity was measured, and renilla luciferase activity was normalized to the firefly luciferase activity. Results are shown as the mean ± SEM and the data are representative of three biological and technical replicates. *p <  0.05, **p <  0.01, n.s., not significant.

To identify the mechanism involved in the miRNA-mediated gene upregulation, we performed 3′ rapid amplification of complementary DNA (cDNA) ends (3' RACE) assay, both in the bovine perirenal and groin fat tissues, and successfully isolated and sequenced a band of 699 bp which aligned downstream of *STK11* coding regions (**Figure 5A**). Interestingly, as indicated in **Figure 5B**, putative RBP binding sequences (GREs and AREs) were found by scanning the entire length of 3' UTR of *STK11*. We postulated that the putative RBP binding sequences located in 3' UTR of *STK11* might be involved in the post-transcriptional regulation of *STK11*.

To evaluate this hypothesis, we constructed dual-luciferase reporter plasmids with or without the putative RBP binding sequences (F-ΔMRE or F-MRE) (illustrated in **Figure 5C**) followed by co-transfection into bovine preadipocytes with miR-424 inhibitor or NC inhibitor. Reporter assays showed that the luciferase activity of the “F-FL” and “F-ΔMRE” reporters was significantly suppressed when miR-424 was absorbed by a specific inhibitor, but resulted in a modest yet significant increase in luciferase activity of the “F-MRE” (**Figure 5D**). These results indicate that the presence of AU/GU rich sequences in the 3' UTR of *STK11* might disrupt the miR-424/*STK11* incorporation into the RNA-induced silencing complexes, suggesting that post-transcriptional regulation of *STK11* by miR-424 occurs in a potential RBP binding site-dependent manner.

## Discussion

It is evident that miRNAs play crucial functions in the regulation of adipogenesis, which provides a solid foundation for the application of miRNA as a molecular marker in the beef production. Recently, we identified the roles of miR-130a/b and miR-23a cluster in the bovine preadipocytes differentiation (Ma et al., 2018; Wang Y. et al., 2018). In the current study, the differentially expressed miRNAs in adult cattle and fetal calf adipose tissues were screened. The results showed that miR-424 was upregulated in adult fat tissue compared to fetal adipose tissue with the highest fold change, which was consistent with our results of miR-424 expression detected in different stages of adipose tissue development. Moreover, we subsequently validated through qRT-PCR that miR-424 gradually increased from the early to middle stage of adipocytes differentiation.

There is a growing consensus that miR-424 exerts a suppressive effect on cell proliferation and cell cycle progression (Liu et al., 2018; Pande et al., 2018; Wang J. et al., 2018). Nonetheless, some studies have indicated that miR-424 could promote cell differentiation by targeting the cell cycle regulator, *Cdc25A*, to enhance cell cycle arrest (Sarkar et al., 2010). miR-424 was also reported to be a strong regulator of monocyte/macrophage differentiation by targeting NFI-A (Rosa et al., 2007; Forrest et al., 2010). Additionally, our previous studies reported high expression level of miR-424 in bovine liver tissues (Yang et al., 2018), suggesting that it might be involved in lipid metabolism in cattle. Hence, in the current study we evaluated the functions and molecular mechanisms of miR-424 in lipogenesis and bovine adipocytes differentiation, and found that the overexpression of miR-424 resulted induced bovine adipocytes differentiation. Therefore, miR-424 appears to have a high hierarchical position among the factors involved in bovine adipogenesis.

One miRNA may simultaneously control multiple adipogenic effector genes, and one adipogenic functional target gene may be bound by several candidate miRNAs. In the current study, the miR-424 target genes (related to adipogenic) were screened out. Notably, we discovered that the endogenous *STK11* expression levels were upregulated when miR-424 was enriched in bovine preadipocytes. A combination of experimental approaches demonstrated that *STK11* was a direct target gene of miR-424. Moreover, we also targeted the regulation of *STK11* transcript through other miRNAs, the dual-luciferase reporter assay showed that the predicted miR-17 (an adipogenic miRNA) as reported previously by Han *et al*. did not directly or physically interacted with *STK11*gene (Han et al., 2017) (**Figure S2**). In addition, our results also showed that specific shRNA against *STK11* could hamper bovine preadipocytes differentiation by decreasing the levels of *CEBP/α*, *PPARγ*, and *FABP4*. Furthermore, the phenotypic effect was proved that miR-424 could not further stimulate adipogenesis in the *STK11*-silent preadipocytes. Therefore, we propose that miR-424 regulates adipogenic differentiation at least in part by activating *STK11*. These results indicate that *STK11* upregulation is necessary for bovine preadipocytes differentiation.

Previously, the regulatory role of *STK11*gene in the lipogenesis was reported in the mice adipose tissue. A white adipose tissue (WAT) mass reduction was observed in *STK11* knockout (FABP4-*STK11*) mice (Zhang et al., 2013). Moreover, deletion of *STK11* and mTOR caused reduction of inguinal white adipose tissue mass (Xiong et al., 2018). The *STK11* also induced white fat browning *via* the PPARγ pathway in 3T3-L1 cell line (Xi et al., 2019). Conversely, our data demonstrates that downregulation of *STK11* suppressed the expression of white preadipocytes differentiation marker genes *C/EBPα, PPARγ,* and *FABP4*) and resulted in a decrease in the accumulation of LDs. It is worth noting that our studies were performed using bovine preadipocytes. It is possible that the role of *STK11* in regulating the development of fat cells is different in different animal models due to the non-conserved nature of the untranslated region of *STK11* gene and the phosphorylation sites of encoded protein in different species.

In addition, recent work has shown that two post-transcriptional pathways of miRNAs and RBPs appear to be involved in glucose homeostasis and lipid metabolism (Kim and Kyung, 2012). Given this data and our current findings, it will be of great interest to determine the exact mechanism of such post-transcriptional upregulation of *STK11* by miR-424. Using the 3' RACE assay, we found that the 3' regulatory region of the bovine *STK11* gene contains miR-424 response elements, and several classical elements, like the GRE (UUGUU) and ARE (AUUUA). We consider the existence of two possible mechanisms of the involvement of RBPs in miR-424 mediated *STK11* upregulation: 1) it is possible that a potential RBP is also a direct target gene for miR-424, and binding to *STK11* indirectly regulates the expression of *STK11*, as in the case of *KSPR* with miR-206 (Amirouche et al., 2014); 2) two types of post-transcriptional regulatory mechanisms exist independently. miR-424 directly regulates *STK11* through the MRE and RBPs directly regulate *STK11* through the ARE or GRE elements, but the latter effect is stronger, such as in the case of *POU1F1* with miR-233 (Wang et al., 2017). However, the identity of the RBPs involved and the specific mechanism of regulation needs to be studied further.

In conclusion, we demonstrate that the pro-adipogenic factor miR-424 regulates bovine preadipocytes through *STK11* in a nonclassical target gene regulation way. Our findings pave the way for a novel candidate molecular marker in the beef production. Moreover, the target *STK11* was identified as a pro-adipogenic factor for bovine preadipocytes differentiation. Our findings also reveal that miR-424 is not conserved among the vertebrates, implying that the miR-424 mediated regulation of *STK11* might be unique to the bovines. To our knowledge, this is the first study to report the role of miR-424 in bovine adipogenesis.

## Data Availability Statement

All data generated or analyzed during this study are included in the article/**Supplementary Material**.

## Ethics Statement

The study was approved by the “Animal Care and Use Committee” of the College of Animal Science and Technology, Northwest A&F University (No. NWAFAC1117).

## Author Contributions

LZ, GC, LW, and SZ conceived and designed the experiments. LW and SZ performed the experiments, analyzed the data, and wrote the manuscript. GC, WZ, ZJ, SL, and RK mainly assisted in providing constructive suggestions for the manuscript and a language modification.

## Funding

This research was supported by the National Key Research Projects (No. 2018YFD0501700), the National Natural Science Foundation of China (NO. 31972994), the National Science and Technology Support Projects (No. 2015BAD03B04), and the National Modern Agricultural Industry Special Program (No. CARS-37).

## Conflict of Interest

The authors declare that the research was conducted in the absence of any commercial or financial relationships that could be construed as a potential conflict of interest.
