# Supplementary material for: miR-424 Promotes Bovine Adipogenesis Through an Unconventional Post-Transcriptional Regulation of STK11
Source: Front Genet. 2020 Mar 4;11:145. doi: 10.3389/fgene.2020.00145 (PMC7064614; doi:10.3389/fgene.2020.00145)
Supplement: Supplementary file 1 [file DataSheet_1.docx]

Supplementary Material

# Supplementary Figures and Table

## Supplementary Figures


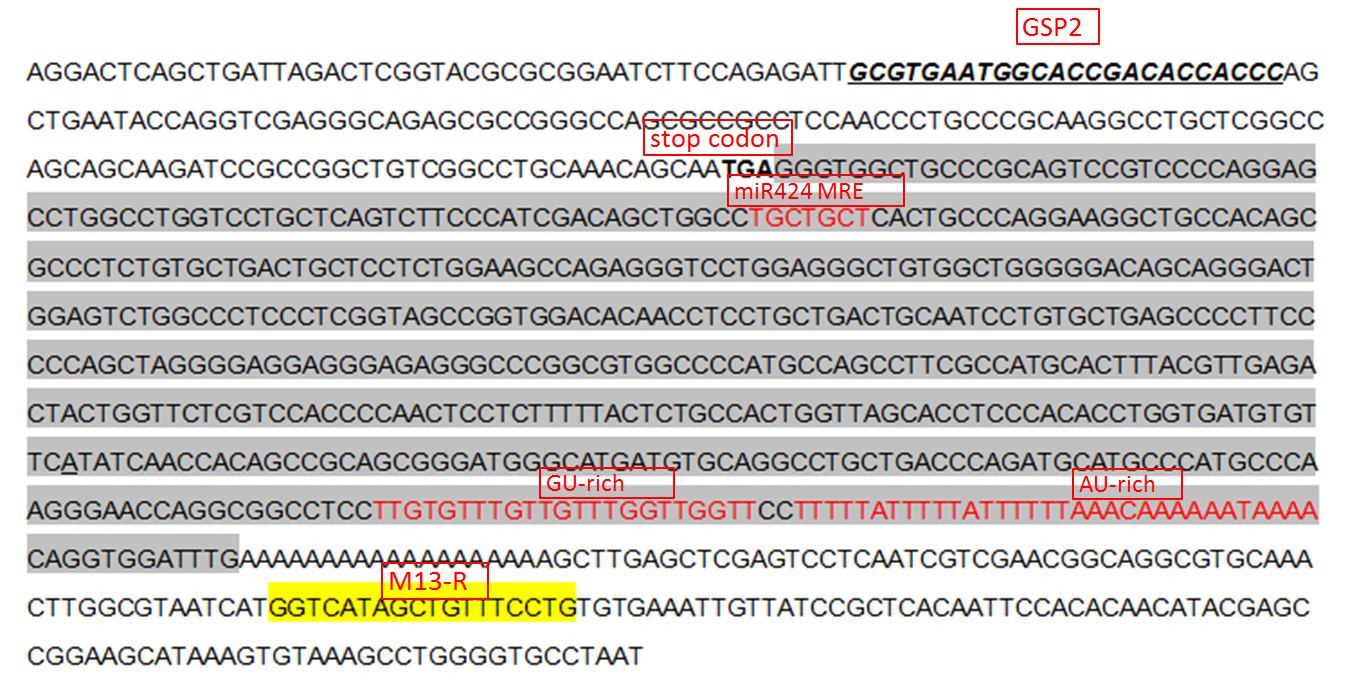


**Supplementary Figure 1.** 3’ untranslated region sequence of the bovine STK11 gene. The gray shadow showes the untranslated region of STK11 gene in bovine fat tissues. The translational end site (TGA) is shown in blood letters. The miR-424-STK11-MRE, GU-rich and AU-rich sequence are shown in red letters, and primer sequences are underlined with the respective names shown below the line. (For interpretation of the references to colour in this figure legend, the reader is referred to the Web version of this article.)


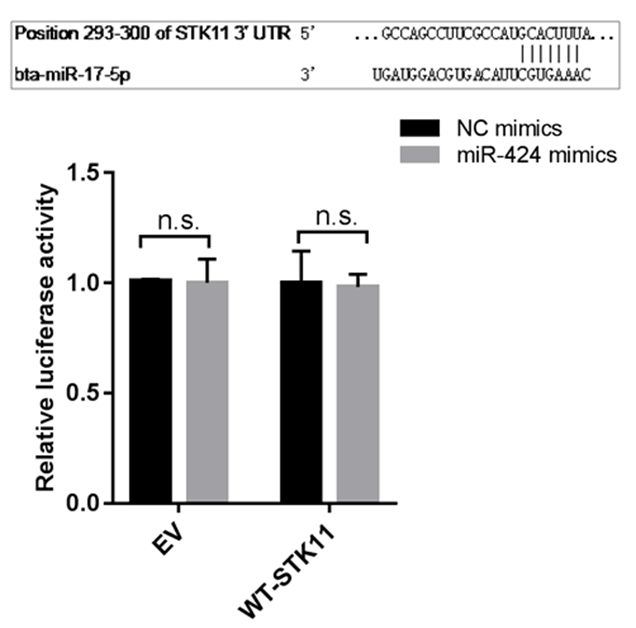


**Supplementary Figure 2.** STK11 is a not direct target of miR-424. (Top) Schematic representation of the sequence of mature miR-17. The putative binding sites were located in the 3' UTR of bovine STK11 gene. (Bottom) Relative luciferase activity was measured in HEK293A cells co-transfected with the psi-Check2 empty vector (EV) or STK11 3' UTR-WT reporter plasmids along with NC mimics or miR-424 mimics. Renilla luciferase activity was normalized to firefly luciferase.

## Supplementary Table

**Supplementary Table1.** Primers sequence information

| **Name** | **Forward Primer (5’-3’)** | **Reversed Primer (5’-3’)** |
| --- | --- | --- |
| **Primer sequences used for real-time PCR** | | |
| bta-miR-424-5p | CAGCAGCAATTCATGTTTTGA | provided by the kit |
| RPS18 | CAGCACACCAAGACCACAG | ACACAAAGCCCACACATTATTTC |
| STK11 | AGTCACGCTCTACAACATCAC | CTGTATGGAGAACCGCTTGG |
| GAPDH | ACACCCTCAAGATTGTCAGC | GTCATAAGTCCCTCCACGAT |
| CEBPα | ATCTGCGAACACGAGACG | CCAGGAACTCGTCGTTGAA |
| CEBPβ | TTCCTCTCCGACCTCTTCTC | CCAGACTCACGTAGCCGTACT |
| PPARγ | TGAAGAGCCTTCCAACTCCC | GTCCTCCGGAAGAAACCCTTG |
| FABP4 | TGAGATTTCCTTCAAATTGGG | CTTGTACCAGAGCACCTTCATC |
| **Primer sequences used for 3’RACE** | | |
| 3'RACE QT | CCAGTGAGCAGAGTGACGAGGACTCGAGCTCAAGCTTTTTTTTTTTTTTTTT | |
| 3'RACE QO | CCAGTGAGCAGAGTGACG | |
| 3'RACE QI | GAGGACTCGAGCTCAAGC | |
| STK11 GSP1 | CCGGCCAAGCGGTTCTCCATACAGC | |
| STK11 GSP2 | GCGTGAATGGCACCGACACCACCC | |
| **Primer sequences used for vector construction** | | |
| F-FL (XhoI/NotI) | C^TCGAGGTCCTGCTCAGTCTTCCCAT | GC^GGCCGCTCAAATCCACCTGTTTTAT |
| F-MRE (XhoI/NotI) | C^TCGAGGTCCTGCTCAGTCTTCCCAT | GC^GGCCGCGAACACATCACCAGGTGTG |
| F-ΔMRE (XhoI/NotI) | C^TCGAGCACACCTGGTGATGTGTTC | GC^GGCCGCTCAAATCCACCTGTTTTAT |
